# Hypoglycemic Encephalopathy With Multisystem Organ Dysfunction in an Infant With MEGD(H)EL Syndrome

**DOI:** 10.7759/cureus.97867

**Published:** 2025-11-26

**Authors:** Brianna Stein, Rylee Simons, Hanna Sahhar, Sami Rishmawi, Anna Kate Shaw

**Affiliations:** 1 Pediatrics, VCOM Carolinas, Spartanburg, USA; 2 Family Medicine, University of Connecticut School of Medicine, Hartford, USA; 3 Pediatric Intensive Care Unit, Spartanburg Regional Healthcare System, Spartanburg, USA; 4 Pediatrics, Clemson University, Clemson, USA

**Keywords:** anoxic brain injury, genetics, hypoglycemic encephalopathy, megd(h)el syndrome, mitochondrial disorder, multisystem organ dysfunction, neurology, pediatrics

## Abstract

This report describes a case of an eight-month-old pediatric patient with 3-methylglutaconic aciduria with deafness-dystonia, hepatopathy, encephalopathy, and Leigh-like syndrome (MEGD(H)EL) syndrome, who initially presented to the emergency room with unresponsiveness and profound hypoglycemia. Laboratory investigations revealed metabolic acidosis, elevated liver function tests, prolonged bleeding times with elevated D-dimer, and a respiratory panel showing human enterovirus/rhinovirus. CSF was positive for human herpesvirus 6. MRI of the brain without contrast revealed relatively bilateral and symmetric diffusion restriction involving the caudate heads, globus pallidi, thalami, ventral cerebral peduncles, and hippocampi. The patient, upon later evaluation, was diagnosed with MEGD(H)EL syndrome. This case highlights the rare syndrome and its unique presentation.

## Introduction

3-methylglutaconic aciduria with deafness-dystonia, hepatopathy, encephalopathy, and Leigh-like syndrome (MEGD(H)EL) syndrome is a rare autosomal recessive disease caused by variants in the serine active site containing 1 (SERAC1) gene. The SERAC1 gene encodes a phospholipid remodeling enzyme that is critical for mitochondrial function. Although the hallmark features such as progressive neurologic deterioration, sensorineural hearing loss, feeding difficulties, and failure to thrive are well-documented, the progression and severity can differ significantly from patient to patient [1]. Definitive diagnosis is established by gene-targeted testing and comprehensive genomic testing. Identification of SERAC1 mutations together with urine organic acid analysis showing high levels of 3-methylglutaconic acid and high urine levels of 3-methylglutaric acid, along with clinical features, can provide a definitive diagnosis for MEGD(H)EL syndrome. Increased urinary excretion of these organic acids is common in inborn errors of metabolism and mitochondrial disorders [2]. We report a healthy, full-term, unvaccinated, eight-month-old male who presented to the hospital for unresponsiveness and was found to have profound hypoglycemia of <10 mg/dL by emergency medical services (EMS). The infant first presented in the pediatric intensive care unit (PICU) with a sepsis-like presentation and hypoglycemia-associated encephalopathy. Subsequent genetic testing at a follow-up visit ultimately established the diagnosis of MEGD(H)EL syndrome. Although MEGD(H)EL syndrome is uncommon, with only a several dozen cases reported worldwide, early recognition is critical due to its rapidly progressive course [1]. This case highlights an atypical early presentation of MEGD(H)EL syndrome in an infant, expanding the known clinical spectrum of this rare mitochondrial disorder. 

## Case presentation

We report an eight-month-old full-term, unvaccinated male patient with no pertinent medical history, who presented to the emergency department via EMS due to unresponsiveness. The patient’s mother reported that the patient had been sick with flu-like symptoms for the past three days, including fever, loss of appetite, and vomiting. She endorsed that his fever broke the day before the emergency department arrival, and the patient had a decreased oral intake. Parents denied any recent travel, sick contacts, or trauma. The patient's mother did state that the patient is solely breastfed and was never given formula or breast milk via bottle. Two pediatric evaluations prior to the emergency department presentation documented poor weight gain, with his weight tracking below the first percentile. EMS reported a blood glucose level of <10 mg/dL (normal range: 70-100 mg/dL) for which he received D10 (dextrose 10%) and glucagon via intraosseous access due to unsuccessful attempts for intravascular access before emergency department arrival. On arrival at the emergency department, his blood sugar was stable at 100 mg/dL. Upon arrival at the emergency department, he was hemodynamically stable and afebrile. On physical examination, the patient was noted to be lethargic and had seizure-like activity. The seizure activity included left gaze deviation and twitching of his right hand, for which he was given lorazepam (Ativan). The patient was admitted to the PICU, where he went through a septic workup, and a lumbar puncture was performed. A femoral central venous catheter was placed and remained in the patient for seven days. Initial laboratory results are summarized in Table 1 below. Notable findings of lab values were markedly elevated liver function tests, indicating hepatocellular injury, severe coagulopathy, lactic acidosis, hypoglycemia with low insulin levels, and significant neurologic injury due to elevated neurofilament light chain. The patient's initial blood gas presented with a pH of 7.29, partial pressure of carbon dioxide of 37 mmHg, partial pressure of oxygen of 49 mmHg, and a bicarbonate of 19 mEq/L, presenting a metabolic acidosis picture. The patient received vitamin K and fresh frozen plasma due to his abnormal coagulation profile, as shown below. The patient's critical initial lab values, in addition to his clinical picture, were consistent with multisystem organ dysfunction. 

**Table 1:** Initial laboratory values

Lab	Patient’s value	Normal range
Respiratory panel	+ Rhino/enterovirus	Negative
CSF analysis	Human herpesvirus 6	Negative
Lactic acid	5.0 millimoles/liter	0.3-2.0 millimoles/liter
Initial beta-hydroxybutyrate	0.05 millimoles/liter	0.00-0.27 millimoles/liter
Calcium	6.8 milligrams/deciliter	8.5-11.0 milligrams/deciliter
White blood cells	25.2 x 10^3/microliter	6.0-17.5 x 10^3/microliter
Alanine transferase	845 international units per liter	8-39 international units per liter
Aspartame transferase	1564 international units per liter	12-33 international units per liter
Alkaline phosphatase	322 international units per liter	110-302 international units per liter
Total bilirubin	4.8 milligrams/deciliter	0.0-1.5 milligrams/deciliter
Prothrombin time	37.2 seconds	12.7-15.1 seconds
International normalized ratio	3.7 seconds	2.0-3.0 seconds
Partial thromboplastin time	99.0 seconds	23.3-36.3 seconds
High-sensitivity troponin	31 picograms per milliliter	<18 picograms per milliliter
Lactate dehydrogenase	811 international units per liter	83-183 international units per liter
Insulin	<0.4 micro-international units per milliliter	2.6-24.9 micro-international units per milliliter
Repeat beta-hydroxybutyrate	0.40 millimoles/liter	0.00-0.27 millimoles/liter
Neurofilament light chain	259 picograms per milliliter	0.00-1.96 picograms per milliliter

**Figure 1:**
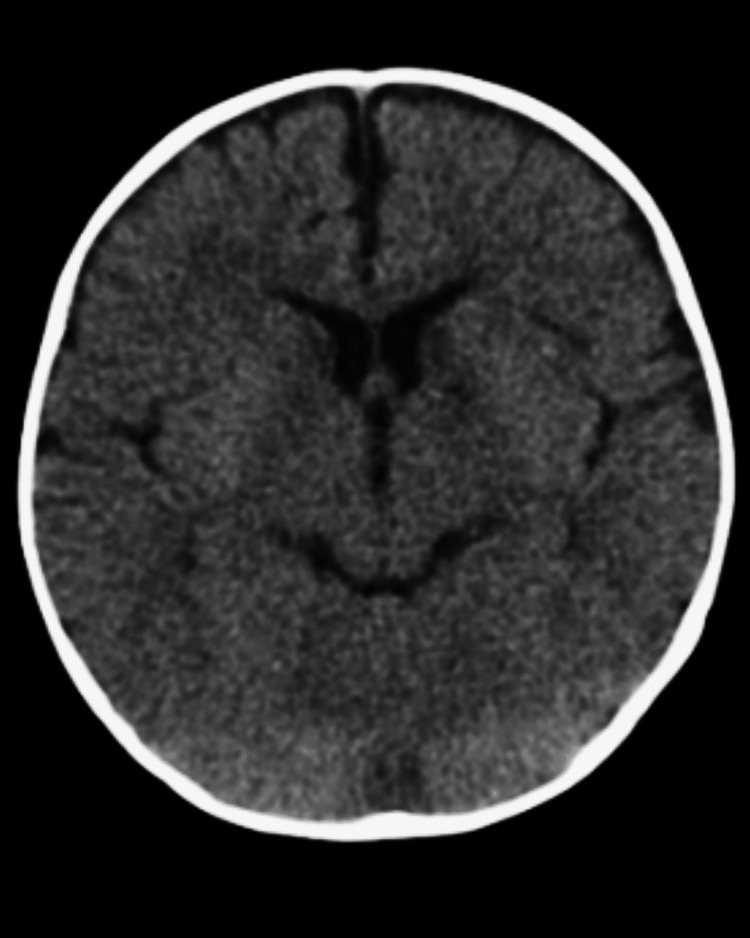
Initial axial non-contrast CT showing no acute intracranial abnormalities.

**Figure 2:**
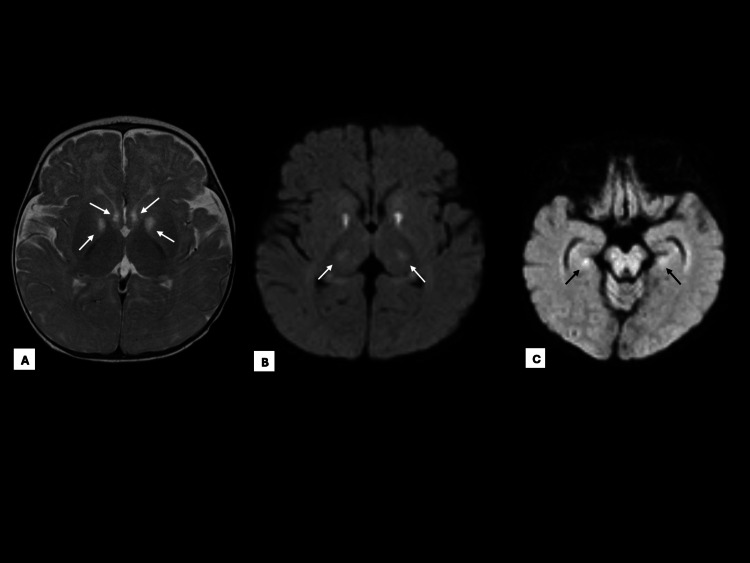
Readout segmentation of long variable echo-trains (RESOLVE) axial T2-diffusion-weighted MRI showing relatively bilateral and symmetric diffusion restriction within the basal ganglia; caudate nucleus and globus pallidus (A), bilateral thalami (B), and hippocampi (C).

**Figure 3:**
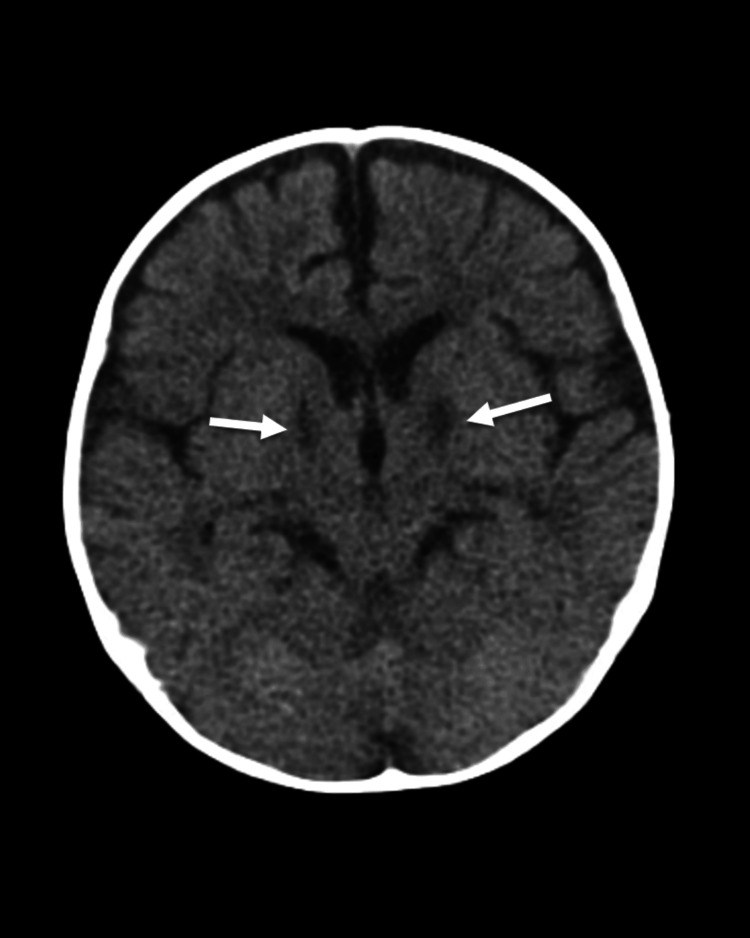
Repeat axial non-contrast CT showing persistent symmetric hypodense foci at the inferior basal ganglia, approximately the genu of the internal capsule, consistent with the MRI appearance. These could be possible small infarcts.

Blood culture and CSF culture showed no growth. Initial non-contrast CT head without contrast showed no acute intracranial abnormality (Figure 1). MRI of the brain without contrast revealed relatively bilateral and symmetric diffusion restriction involving the caudate heads, globus pallidi, thalami, ventral cerebral peduncles, and hippocampi (Figure 2). The cerebellum, brainstem, ventricular system, and craniocervical junction were unremarkable. There was no involvement of the cerebral cortex or splenium of the corpus callosum. Repeat CT imaging on day four of admission revealed no new abnormalities and some possible improvement of the gray/white matter differentiation at the hemispheres when compared to the MRI, but the differentiation was still somewhat diminished. Additionally, the CT showed persistent symmetric hypodense foci at the inferior basal ganglia, approximately the genu of the internal capsule, consistent with the MRI appearance, which could be possible small infarcts (Figure 3). Electroencephalogram showed moderate diffuse slowing of background rhythms consistent with diffuse encephalopathy, but no focal slowing or epileptiform activity.

During admission, the patient became hypoglycemic again with a blood glucose of 42 mg/dL. A critical sample for hypoglycemia was drawn, including insulin, C-peptide, free fatty acids, growth hormone, cortisol, ammonia, lactate, beta-hydroxybutyrate, acylcarnitine profile, free and total carnitine, and urine organic acids (Table 2). Significant results included elevated lactate dehydrogenase, decreased insulin, and mildly elevated beta-hydroxybutyrate, a pattern that is highly consistent with a metabolic crisis due to an underlying mitochondrial disorder rather than hyperinsulinemia. While in the PICU, the patient received occupational, speech, and physical therapy. Feedings via nasogastric tube were initiated due to aphagia and risk of aspiration. Upon discharge, the patient fully tolerated oral milk via breastfeeding but was still hypotonic and had an occasional high-pitched cry. He was referred to outpatient occupational, speech, and physical therapy, as well as pediatric neurology.

**Table 2:** Critical hypoglycemia profile

Test	Patient’s value	Normal range
Lactate dehydrogenase	811 international units per liter	83-183 international units per liter
Insulin	<0.4 micro-international units per milliliter	2.6-24.9 micro-international units per milliliter
Beta-hydroxybutyrate	0.40 millimoles/liter	0.00-0.27 millimoles/liter
Urine organic acids: tyrosine metabolites	Elevated (4-hydroxyphenyllactate, 4-hydroxyphenylpyruvate)	Normal: not elevated
Urine organic acids: 3-methylglutaconic acid	Moderate elevation	Normal: not elevated
Urine organic acids: 3-methylglutaric acid	Moderate elevation	Normal: not elevated
Succinylacetone	Not detected	Absent
Neurofilament light chain	259 picograms per milliliter	0.00-1.96 picograms per milliliter

At the age of 13 months, the patient was referred to genetic counseling due to his failure to thrive and previous abnormal newborn screenings, including elevated tyrosine at 30 hours of life and elevated total galactose at 250 hours of life, raising concern for an inborn error in metabolism. It was revealed that the patient’s brother had a confirmed diagnosis of MEGD(H)EL and another variant of uncertain significance; parental testing was not completed. Laboratory testing was performed on the infant, which included targeted analysis of the SERAC 1 gene showing a pathogenic homozygous deletion/insertion alteration at nucleotide 1822 of the SERAC1 gene and galactose-1-phosphate (Gal-1-p). Gal-1-p was within normal limits, but urine organic acid analysis revealed moderate elevations of tyrosine metabolites (4-hydroxyphenyllactate and 4-hydroxyphenylpyruvate), as well as 3-methylglutaconic acid and 3-methylglutaric acid. At the patient's recent visit to the geneticist on April 1, 2025, the patient exhibited developmental regression and was no longer able to sit unsupported. The patient had truncal hypotonia with hypertonia noted in the distal extremities upon examination. The patient kept his hands clenched bilaterally. The patient made noises, but no words or sentences. The patient was also referred to ophthalmology and audiology for further evaluation.

## Discussion

MEGD(H)EL syndrome is a rare autosomal recessive condition caused by mutations in the serine active site containing 1 (SERAC1) gene on chromosome 6q25.3 [3]. The SERAC1 gene encodes a phosphatidylglycerol (PG) remodeling enzyme located at the mitochondria and endoplasmic reticulum interface, where it plays a critical role in mitochondrial phospholipid remodeling, specifically the conversion of PG into cardiolipin, a phospholipid essential for mitochondrial membrane stability [4]. These genetic and metabolic disruptions underlie the characteristic combination of metabolic, hepatic, and neurologic abnormalities observed in MEGD(H)EL syndrome.

It is estimated that 27 patients per year are born with MEGD(H)EL syndrome worldwide [1]. The median age at diagnosis was 7.2 years, although the clinical and phenotypic spectrum ranges from early infancy to adulthood [5,6]. Infantile onset commonly presents with hypoglycemia and a sepsis-like clinical presentation without an infectious cause. Additional symptoms can present in an infant as feeding difficulties due to dysphagia and drooling, failure to thrive, progressive sensorineural hearing loss, diffuse muscle hypotonia, and liver involvement. Adult onset can be milder in manifestations and show generalized dystonia [7]. As the patient ages, neurologic symptoms become more evident. These can present as spasticity, dystonia, lack of the development of speech, and hypoacusis [2].

Biochemical abnormalities in MEGD(H)EL reflect the underlying mitochondrial and phospholipid trafficking dysfunction. Elevations of 4-hydroxyphenyllactate and 4-hydroxyphenylpyruvate can be associated with liver immaturity/dysfunction or intestinal bacteria metabolism. These values can be a transient finding in a neonate, especially in a premature infant. Elevated excretion of 3-methylglutaconic acid and 3-methylglutaric acid is a biochemical finding of difficult interpretation, as 3-methylglutaconic aciduria is a clinically and genetically heterogeneous group of metabolic disorders. Nonetheless, a laboratory finding of elevated urinary concentration of 3-methylglutaconic acid and 3-methylglutaric acid on routine analysis of urine organic acids is a biochemical hallmark of MEGD(H)EL syndrome. In addition, serum lactate concentration and alanine concentration can be elevated but are non-specific to MEGD(H)EL. A normal control for urinary concentration of 3-methylglutaconic acid is under 10 mmol/mol creatinine in healthy individuals. 

Radiologic findings can offer further diagnostic support of MEGD(H)EL syndrome. MRI findings of MEGD(H)EL syndrome will show signs of atrophy to the basal ganglia, putamen, cerebrum, and cerebellum [6,8,9]. In our patient, atrophy to the basal ganglia correlates with clinical features of difficulty feeding, rigidity with clenched fists, and hypotonia. Cerebrum and cerebellum atrophy are consistent with his developmental delay, poor coordination, hypotonia, and poor speech development noted at his most recent follow-up visit. A five-year-old patient with hypotonia, hyperlaxity, and hyperreflexia received an MRI with bilateral symmetric anterior putamen and caudate abnormal signal intensity, indicating caudate atrophy and symmetric abnormal signal intensity in the basal ganglia. After whole exome testing showed a mutation in the SERAC1 gene on chromosome 16, the patient was diagnosed with MEGD(H)EL syndrome. This MRI presentation resembles the pattern of findings observed in our infants' MRI and CT radiologic images [10].

The clinical presentation of MEGD(H)EL syndrome is highly variable, which makes early diagnosis challenging [2]. Although the hallmark features are well-documented, including the characteristic combination of metabolic, neurological, and hepatic symptoms, the progression and severity of this disease can differ significantly from patient to patient. The disease progresses as the patients age; additional complications, including cognitive impairment, epilepsy, limited communication, scoliosis, and progressive deafness, arise due to ongoing mitochondrial dysfunction and damage to the cochlea [2,9]. Given the progressive hearing loss in these patients, most do not achieve spoken language or communication skills [11]. Given the complexity of the syndrome, a multidisciplinary approach involving therapies to work on strength, speech therapy for communication, neurology, audiology, ophthalmology, and supportive care is essential for managing affected individuals [1]. These patients are dependent on a caregiver for their activities of daily living and are wheelchair bound.

Our patient demonstrates an unusual example of a previously healthy, unvaccinated infant presenting with profound hypoglycemia leading to encephalopathy, anoxic brain injury, and multisystem organ failure. Furthermore, we present the unique MRI findings of relatively bilateral and symmetric diffusion restriction involving the caudate heads, globus pallidi, thalami, ventral cerebral peduncles, and hippocampi, without involvement of the cortex or corpus callosum, which are consistent with the Leigh-like neuroimaging pattern described in MEGD(H)EL syndrome [12]. These findings demonstrate the unique clinic vignette of MEGD(H)EL syndrome with dystonia, hepatopathy, encephalopathy with ischemic brain injury, seizures, and aphagia. Targeted genetic testing of our patient confirmed a pathogenic homozygous deletion/insertion at nucleotide 1822 of the SERAC1 gene, corroborating the biochemical, radiologic, and clinical findings of MEGD(H)EL syndrome in the infant. Due to our patient and his brother both acquiring this rare autosomal recessive genetic condition, it is confirmed that their parents are genetic carriers for MEGD(H)EL.

Further research is needed to assess the best treatment management for patients with MEGD(H)EL syndrome and to determine whether alternative therapies can be used to help prevent the worsening or progression of this disease. Finally, neurodevelopmental predictions from studies focused on neonatal encephalopathy may not reliably reflect the long-term deficits for our infant due to the distinctive and progressive nature of MEGD(H)EL syndrome.

## Conclusions

In conclusion, a patient presenting with a sepsis-like picture, profound hypoglycemia, and diffuse muscle hypotonia with no infectious source should prompt recognition and evaluation of a genetic mitochondrial disorder with concurrent lab testing. Early involvement with specialists, including physical therapists, audiologists, neurologists, ophthalmologists, and geneticists, is vital for continued survival and optimizing long-term care success of patients with MEGD(H)EL syndrome. Further research of the disease process should be conducted to evaluate if genetic modifying treatments can improve these patients' outcomes and prevent the worsening of the disease processes.
